# Benzene-1,3,5-tri­carb­oxy­lic acid–pyridinium-2-olate (1/3)

**DOI:** 10.1107/S1600536814005534

**Published:** 2014-03-19

**Authors:** José J. Campos-Gaxiola, Felipe Zamora Falcon, Ramón Corral Higuera, Herbert Höpfl, Adriana Cruz-Enríquez

**Affiliations:** a Facultad de Ingenieria Mochis, Universidad Autónoma de Sinaloa, Fuente Poseidón y Prol. A. Flores S/N, CP 8122, C.U. Los Mochis, Sinaloa, México; b Centro de Investigaciones Quimicas, Universidad Autónoma del Estado de Morelos, Av. Universidad 1001, CP 62210, Cuernavaca, Morelos, México

## Abstract

The asymmetric unit of the title compound, C_9_H_6_O_6_·3C_5_H_5_NO, contains one benzene-1,3,5-tri­carb­oxy­lic acid mol­ecule (BTA) and three pyridin-2-ol mol­ecules each present in the zwitterion form. In the crystal, these entities are linked through O—H⋯O^−^ and N^+^—H⋯O^−^ hydrogen bonds, forming sheets parallel to (10-1). These layers contain macrocyclic rings of composition [BTA]_2_[pyol]_6_ and with graph-set notation *R* ^6^_8_(44), which are stacked along *c* through π–π inter­actions [inter-centroid distances = 3.536 (2)–3.948 (3) Å]. They are inter­connected by N^+^—H⋯O^−^ hydrogen-bonded chains of pyridin-2-ol mol­ecules running parallel to *c*, forming a three-dimensional network. There are also C—H⋯O hydrogen bonds present which reinforce the three-dimensional structure.

## Related literature   

For reports on supra­molecular crystal engineering and potential applications of co-crystals, see: Desiraju (1995 ▶); Karki *et al.* (2009 ▶); Aakeröy *et al.* (2010 ▶); Yan *et al.* (2012 ▶); Li *et al.* (2014 ▶); Ebenezer & Mu­thiah (2012 ▶. For background to related crystal structures, see: Bhogala *et al.* (2005 ▶); Shattock *et al.* (2008 ▶); Yu (2012 ▶).
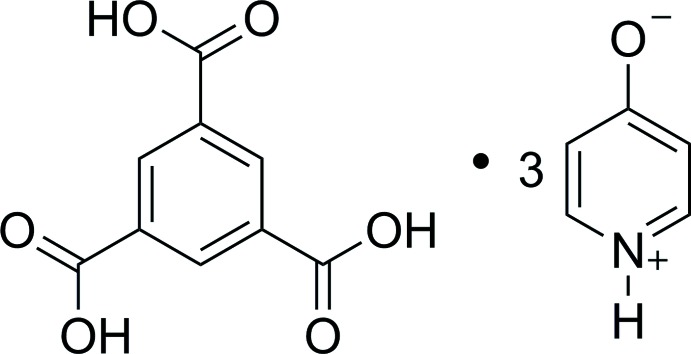


## Experimental   

### 

#### Crystal data  

C_9_H_6_O_6_·3C_5_H_5_NO
*M* *_r_* = 495.44
Monoclinic, 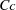

*a* = 14.344 (2) Å
*b* = 25.993 (5) Å
*c* = 6.7047 (10) Å
β = 117.472 (2)°
*V* = 2217.8 (6) Å^3^
*Z* = 4
Mo *K*α radiation
μ = 0.12 mm^−1^
*T* = 100 K
0.49 × 0.41 × 0.34 mm

#### Data collection  

Bruker APEX CCD area-detector diffractometer
Absorption correction: multi-scan (*SADABS*; Sheldrick, 1996 ▶) *T*_min_ = 0.95, *T*_max_ = 0.96
12173 measured reflections
2433 independent reflections
2354 reflections with *I* > 2σ(*I*)
*R*_int_ = 0.076

#### Refinement  

*R*[*F* ^2^ > 2σ(*F* ^2^)] = 0.047
*wR*(*F* ^2^) = 0.104
*S* = 1.08
2433 reflections
343 parameters
8 restraints
H atoms treated by a mixture of independent and constrained refinement
Δρ_max_ = 0.33 e Å^−3^
Δρ_min_ = −0.25 e Å^−3^

### 

Data collection: *SMART* (Bruker, 2001 ▶); cell refinement: *SAINT-Plus-NT* (Bruker 2001 ▶); data reduction: *SAINT-Plus-NT*; program(s) used to solve structure: *SHELXS97* (Sheldrick, 2008 ▶); program(s) used to refine structure: *SHELXL97* (Sheldrick, 2008 ▶); molecular graphics: *ORTEP-3 for Windows* (Farrugia, 2012 ▶) and *Mercury* (Macrae *et al.*, 2008 ▶); software used to prepare material for publication: *SHELXTL* (Sheldrick, 2008 ▶) and *publCIF* (Westrip, 2010 ▶).

## Supplementary Material

Crystal structure: contains datablock(s) I, New_Global_Publ_Block. DOI: 10.1107/S1600536814005534/su2712sup1.cif

Structure factors: contains datablock(s) I. DOI: 10.1107/S1600536814005534/su2712Isup2.hkl

Supporting information file. DOI: 10.1107/S1600536814005534/su2712Isup3.cml

CCDC reference: 991203

Additional supporting information:  crystallographic information; 3D view; checkCIF report

## Figures and Tables

**Table 1:** Hydrogen-bond geometry (Å, °)

*D*—H⋯*A*	*D*—H	H⋯*A*	*D*⋯*A*	*D*—H⋯*A*
O1—H1′⋯O8^i^	0.84	1.72	2.555 (3)	173
O3—H3′⋯O7^ii^	0.84	1.70	2.489 (3)	156
O5—H5′⋯O9^iii^	0.84	1.70	2.531 (4)	170
N1—H1*A*⋯O7^iv^	0.84	1.91	2.712 (4)	158
N2—H2*A*⋯O8^v^	0.84	2.00	2.817 (4)	165
N3—H3*A*⋯O9^vi^	0.84	2.09	2.825 (4)	146
C14—H14⋯O6	0.95	2.67	3.596 (5)	166
C19—H19⋯O2^vii^	0.95	2.48	3.034 (5)	117
C24—H24⋯O4^viii^	0.95	2.45	3.067 (6)	123
C13—H13⋯O9^iii^	0.95	2.63	3.270 (4)	125
C10—H10⋯O3^ix^	0.95	2.42	3.073 (5)	126
C16—H16⋯O1^x^	0.95	2.68	3.307 (4)	124
C19—H19⋯O6^xi^	0.95	2.57	3.314 (6)	135
C20—H20⋯O6^xii^	0.95	2.55	3.499 (4)	176
C23—H23⋯O4^xiii^	0.95	2.48	3.310 (4)	147

Symmetry codes: (i) 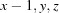; (ii) 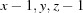; (iii) 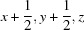; (iv) 
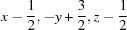
; (v) 
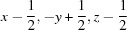
; (vi) 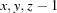; (vii) 
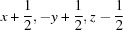
; (viii) 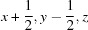; (ix) 
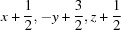
; (x) 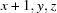; (xi) 
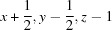
; (xii) 
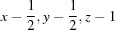
; (xiii) 
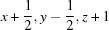
.

**Fig. 1.**
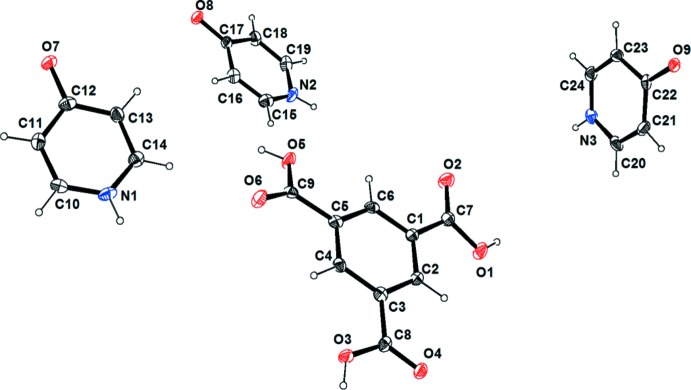
The molecular structure of the title compound, with atom labelling. The displacement ellipsoids are drawn at the 50% probability level.

**Fig. 2.**
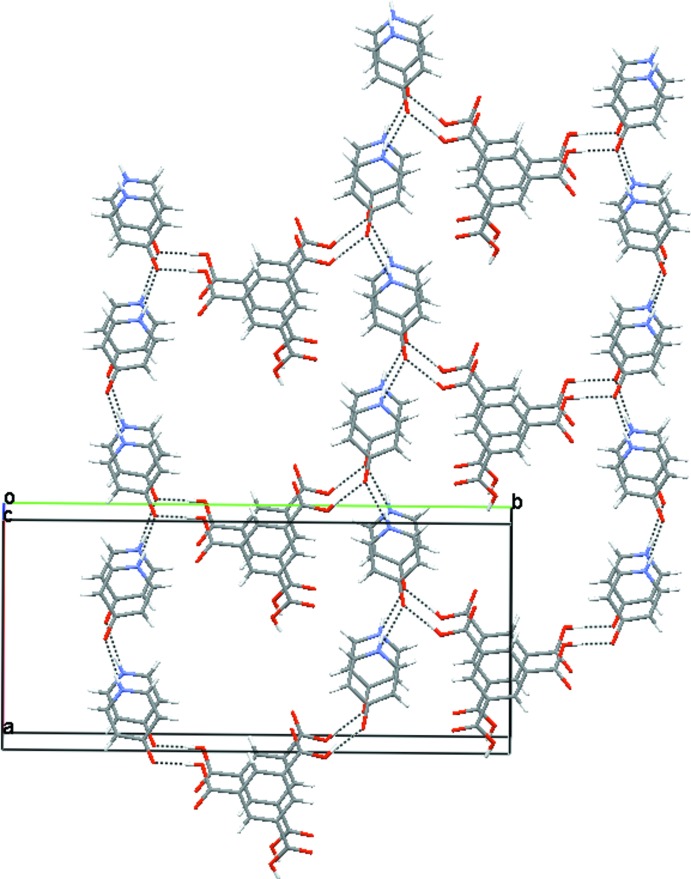
A view along the a axis of the crystal packing of the title compound, showing the two-dimensional hydrogen bonded sheets parallel to (10-1) (see Table 1 for details).

**Fig. 3.**
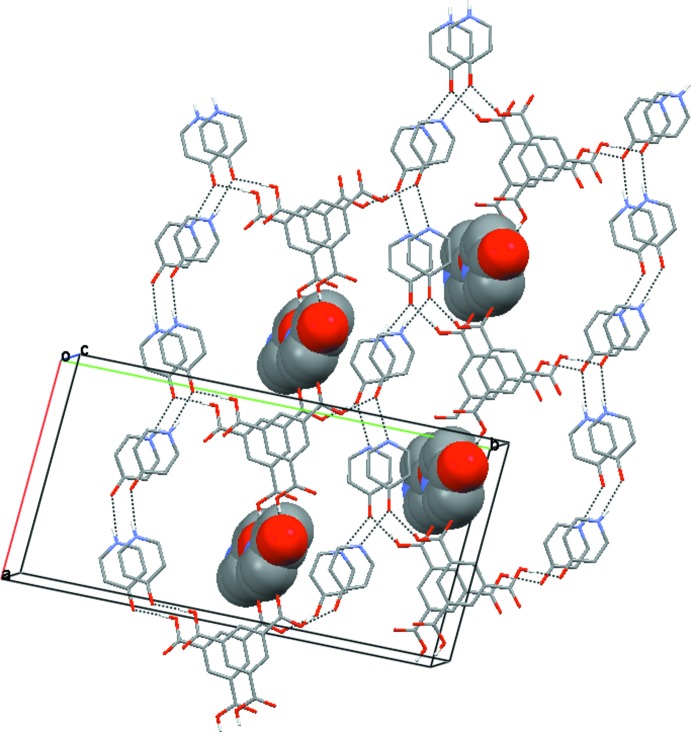
A view along the a axis of the crystal packing of the title compound, showing the three-dimensional hydrogen bonded network formed through O—H···-O and N+—H···-O hydrogen bonds (see Table 1 for details; H atoms not involved in hydrogen bonding have been omitted for clarity).

## supplementary crystallographic information

### 1. Comment

The engineering and design of novel materials *via* non-covalent synthesis has developed as a very attractive and potential area of research because of its importance in molecular recognition (Aakeröy *et al.*, 2010; Li *et al.*, 2014), pharmaceutical chemistry (Karki *et al.*, 2009) and materials chemistry (Yan *et al.*, 2012). Aromatic carboxylic acids form reliable supramolecular synthons for the construction of novel organic networks by hydrogen bonding and π–π interactions (Desiraju, 1995), and numerous studies have focused on hydrogen bonding between carboxylic acids and pyridine derivatives (Bhogala *et al.*, 2005; Shattock *et al.* 2008; Yu, 2012). Herein, we report on the solid-state structure of a 1:3 co-crystal formed between benzene-1,3,5-tricarboxylic acid and pyridin-2-ol.

The asymmetric unit of the title compound contains one benzene-1,3,5-tricarboxylic acid and three pyridin-2-ol molecules in the zwitterion form (Fig. 1). In the benzene-1,3,5-tricarboxylic acid (BTA) molecule, the mean planes of the three carboxyl groups are twisted by 3.9 (2), 9.3 (2) and 13.3 (2)° relative to the benzene ring mean plane.

In the crystal lattice, the BTA molecules and two of the three independent zwitterionic pyridine-2ol entities are linked through O—H···^-^O and N^+^—H···^-^O hydrogen bonds into two-dimensional hydrogen bonded layers parallel to (10-1) (see Table 1 and Fig. 2). These two-dimensional sheets are stacked through π–π interactions along *c* and interpenetrated by one-dimensional hydrogen bonded chains formed by the third group of independent pyridin-2-ol molecules, through N^+^—H···^-^O hydrogen bonds, giving an overall three-dimensional hydrogen bonded skeleton (Table 1 and Fig. 3). The supramolecular network is further accomplished by C—H···O hydrogen bonds (Table 1). Strong π–π interactions are formed between the BTA molecules [*Cg*1···*Cg*1^i^ = 3.536 (2) Å; *Cg*1 centroid of ring C1—C6; symmetry code: (i) *x*, -*y*+1, *z* - 1/2]. There and also weaker π–π interactions present involving two of the three independent pyridin-2-ol entities [*Cg*2···*Cg*3^ii^ = 3.921 (2) and *Cg*2···*Cg*3^iii^ = 3.948 (3) Å; *Cg*2 centroid of ring N1/C10—C14, *Cg*3 centroid of ring N2/C15—C19; symmetry codes:(ii) *x*, -*y*+1, *z* + 3/2; (iii) *x*, -*y*+1, *z*+1/2].

### 2. Experimental

A solution of 4-hydroxypyridine (0.050 g, 0.525 mmol) and benzene-1,3,5-tricarboxylic acid (0.055 g, 0.262 mmol) in a solvent mixture of THF and DMF (7.5 ml, 2:1, *v*/*v*) was stirred for 30 min at room temperature, giving a clear transparent solution. Upon slow evaporation of the solvents during approximately 30 days, yellow crystals were obtained. Spectroscopic data for the title compound are available in the archived CIF.

### 3. Refinement

The C-bound H atoms were positioned geometrically and treated as riding atoms: C—H = 0.95 Å with *U*_iso_(H) = 1.2*U*_eq_(C). The H atoms bonded to O and N were initially located in a difference Fourier map. They were refined with an *X*—H distance restraint of 0.840 (1) Å with *U*_iso_(H) = 1.5*U*_eq_(O,N).

### Crystal data

**Table:**

C_9_H_6_O_6_·3C_5_H_5_NO	*F*(000) = 1032
*M**_r_* = 495.44	*D*_x_ = 1.484 Mg m^−^^3^
Monoclinic, *C**c*	Mo *K*α radiation, λ = 0.71073 Å
Hall symbol: C -2yc	Cell parameters from 4866 reflections
*a* = 14.344 (2) Å	θ = 2.8–28.5°
*b* = 25.993 (5) Å	µ = 0.12 mm^−^^1^
*c* = 6.7047 (10) Å	*T* = 100 K
β = 117.472 (2)°	Rectangular prism, colorless
*V* = 2217.8 (6) Å^3^	0.49 × 0.41 × 0.34 mm
*Z* = 4	

### Data collection

**Table:**

Bruker APEX CCD area-detector diffractometer	2433 independent reflections
Radiation source: fine-focus sealed tube	2354 reflections with *I* > 2σ(*I*)
Graphite monochromator	*R*_int_ = 0.076
phi and ω scans	θ_max_ = 27.0°, θ_min_ = 1.8°
Absorption correction: multi-scan (*SADABS*; Sheldrick, 1996)	*h* = −18→18
*T*_min_ = 0.95, *T*_max_ = 0.96	*k* = −33→32
12173 measured reflections	*l* = −8→8

### Refinement

**Table:**

Refinement on *F*^2^	Primary atom site location: structure-invariant direct methods
Least-squares matrix: full	Secondary atom site location: difference Fourier map
*R*[*F*^2^ > 2σ(*F*^2^)] = 0.047	Hydrogen site location: inferred from neighbouring sites
*wR*(*F*^2^) = 0.104	H atoms treated by a mixture of independent and constrained refinement
*S* = 1.08	*w* = 1/[σ^2^(*F*_o_^2^) + (0.0394*P*)^2^ + 2.5063*P*] where *P* = (*F*_o_^2^ + 2*F*_c_^2^)/3
2433 reflections	(Δ/σ)_max_ < 0.001
343 parameters	Δρ_max_ = 0.33 e Å^−^^3^
8 restraints	Δρ_min_ = −0.25 e Å^−^^3^

### Special details

**Table:**

Experimental. Spectroscopic data for the title compound: IR (KBr, cm^-1^): 3442, 3103, 3050, 1704, 1690, 1624, 1613, 1468, 1282, 1193, 1094, 1024.
Geometry. All e.s.d.'s (except the e.s.d. in the dihedral angle between two l.s. planes) are estimated using the full covariance matrix. The cell e.s.d.'s are taken into account individually in the estimation of e.s.d.'s in distances, angles and torsion angles; correlations between e.s.d.'s in cell parameters are only used when they are defined by crystal symmetry. An approximate (isotropic) treatment of cell e.s.d.'s is used for estimating e.s.d.'s involving l.s. planes.
Refinement. Refinement of *F*^2^ against ALL reflections. The weighted *R*-factor *wR* and goodness of fit *S* are based on *F*^2^, conventional *R*-factors *R* are based on *F*, with *F* set to zero for negative *F*^2^. The threshold expression of *F*^2^ > σ(*F*^2^) is used only for calculating *R*-factors(gt) *etc*. and is not relevant to the choice of reflections for refinement. *R*-factors based on *F*^2^ are statistically about twice as large as those based on *F*, and *R*– factors based on ALL data will be even larger.

### Fractional atomic coordinates and isotropic or equivalent isotropic displacement parameters (Å^2^)

**Table:**

	*x*	*y*	*z*	*U*_iso_*/*U*_eq_	
N1	0.5716 (2)	0.74048 (12)	0.7948 (5)	0.0185 (7)	
H1A	0.5074 (9)	0.7479 (18)	0.734 (7)	0.028*	
N2	0.7381 (2)	0.23549 (13)	−0.0482 (5)	0.0212 (7)	
H2A	0.6809 (18)	0.2201 (16)	−0.086 (8)	0.032*	
N3	0.1812 (2)	0.06645 (12)	0.3136 (5)	0.0183 (6)	
H3A	0.191 (3)	0.0665 (17)	0.199 (4)	0.027*	
O1	0.0316 (2)	0.39495 (9)	0.3690 (5)	0.0200 (5)	
H1'	0.031 (4)	0.3628 (2)	0.355 (8)	0.030*	
O2	0.2044 (2)	0.38226 (10)	0.5822 (5)	0.0214 (6)	
O3	−0.00333 (19)	0.63687 (10)	0.3527 (4)	0.0196 (6)	
H3'	−0.053 (2)	0.6581 (13)	0.294 (7)	0.029*	
O4	−0.11698 (19)	0.57121 (9)	0.2541 (4)	0.0164 (5)	
O5	0.43466 (18)	0.53127 (10)	0.7273 (4)	0.0183 (5)	
H5'	0.4927 (17)	0.5446 (16)	0.755 (8)	0.027*	
O6	0.3814 (2)	0.61071 (10)	0.7524 (5)	0.0218 (6)	
O7	0.8839 (2)	0.71094 (10)	1.1439 (4)	0.0180 (5)	
O8	1.02950 (19)	0.29837 (9)	0.2960 (4)	0.0183 (5)	
O9	0.11946 (18)	0.06361 (10)	0.8481 (4)	0.0152 (5)	
C1	0.1406 (3)	0.46845 (14)	0.5090 (6)	0.0129 (7)	
C2	0.0532 (3)	0.50081 (13)	0.4269 (5)	0.0113 (6)	
H2	−0.0156	0.4866	0.3620	0.014*	
C3	0.0669 (3)	0.55406 (14)	0.4403 (6)	0.0127 (7)	
C4	0.1686 (3)	0.57493 (13)	0.5353 (6)	0.0127 (7)	
H4	0.1783	0.6112	0.5452	0.015*	
C5	0.2549 (3)	0.54226 (13)	0.6147 (5)	0.0125 (7)	
C6	0.2412 (3)	0.48950 (13)	0.6015 (6)	0.0136 (7)	
H6	0.3008	0.4675	0.6558	0.016*	
C7	0.1295 (3)	0.41098 (14)	0.4918 (6)	0.0150 (7)	
C8	−0.0277 (3)	0.58794 (13)	0.3392 (6)	0.0124 (7)	
C9	0.3641 (3)	0.56526 (13)	0.7065 (6)	0.0134 (7)	
C10	0.6414 (3)	0.77853 (13)	0.8312 (6)	0.0146 (7)	
H10	0.6164	0.8119	0.7733	0.017*	
C11	0.7469 (3)	0.77035 (14)	0.9492 (6)	0.0160 (7)	
H11	0.7947	0.7979	0.9756	0.019*	
C12	0.7854 (3)	0.72007 (14)	1.0328 (6)	0.0156 (7)	
C13	0.7089 (3)	0.68087 (13)	0.9850 (6)	0.0161 (7)	
H13	0.7308	0.6467	1.0352	0.019*	
C14	0.6033 (3)	0.69193 (15)	0.8667 (6)	0.0182 (7)	
H14	0.5527	0.6655	0.8355	0.022*	
C15	0.7479 (3)	0.28586 (15)	0.0073 (6)	0.0203 (8)	
H15	0.6867	0.3060	−0.0321	0.024*	
C16	0.8446 (3)	0.30826 (14)	0.1192 (6)	0.0188 (7)	
H16	0.8495	0.3439	0.1542	0.023*	
C17	0.9382 (3)	0.27898 (14)	0.1843 (6)	0.0163 (7)	
C18	0.9227 (3)	0.22593 (14)	0.1212 (7)	0.0195 (8)	
H18	0.9820	0.2044	0.1594	0.023*	
C19	0.8238 (3)	0.20564 (15)	0.0069 (6)	0.0216 (8)	
H19	0.8153	0.1703	−0.0341	0.026*	
C20	0.0841 (3)	0.07837 (14)	0.2819 (6)	0.0189 (8)	
H20	0.0307	0.0861	0.1349	0.023*	
C21	0.0613 (3)	0.07946 (14)	0.4590 (6)	0.0183 (7)	
H21	−0.0070	0.0890	0.4347	0.022*	
C22	0.1399 (3)	0.06629 (13)	0.6804 (6)	0.0142 (7)	
C23	0.2420 (3)	0.05643 (13)	0.7041 (6)	0.0162 (7)	
H23	0.2985	0.0498	0.8488	0.019*	
C24	0.2592 (3)	0.05641 (13)	0.5203 (6)	0.0178 (7)	
H24	0.3276	0.0492	0.5386	0.021*	

### Atomic displacement parameters (Å^2^)

**Table:**

	*U*^11^	*U*^22^	*U*^33^	*U*^12^	*U*^13^	*U*^23^
N1	0.0100 (15)	0.0280 (17)	0.0156 (15)	0.0043 (13)	0.0042 (13)	−0.0001 (13)
N2	0.0156 (16)	0.0321 (19)	0.0161 (15)	−0.0092 (13)	0.0075 (13)	−0.0042 (13)
N3	0.0239 (17)	0.0187 (15)	0.0168 (16)	−0.0008 (12)	0.0133 (14)	0.0001 (12)
O1	0.0166 (13)	0.0125 (11)	0.0273 (14)	−0.0030 (10)	0.0070 (11)	−0.0028 (11)
O2	0.0153 (13)	0.0174 (13)	0.0278 (14)	0.0012 (10)	0.0068 (11)	0.0000 (11)
O3	0.0109 (12)	0.0159 (12)	0.0276 (14)	0.0039 (10)	0.0050 (11)	0.0045 (11)
O4	0.0122 (12)	0.0176 (12)	0.0200 (13)	−0.0002 (10)	0.0080 (10)	0.0008 (10)
O5	0.0093 (12)	0.0201 (13)	0.0260 (14)	−0.0011 (10)	0.0086 (11)	−0.0021 (11)
O6	0.0125 (12)	0.0207 (14)	0.0291 (15)	−0.0026 (10)	0.0069 (11)	−0.0036 (11)
O7	0.0101 (12)	0.0183 (13)	0.0212 (13)	−0.0004 (9)	0.0035 (10)	0.0010 (10)
O8	0.0121 (12)	0.0149 (12)	0.0237 (13)	−0.0007 (9)	0.0047 (11)	−0.0006 (10)
O9	0.0113 (12)	0.0231 (13)	0.0121 (12)	−0.0027 (10)	0.0062 (10)	−0.0014 (10)
C1	0.0130 (17)	0.0189 (17)	0.0074 (15)	−0.0004 (13)	0.0051 (13)	−0.0004 (12)
C2	0.0080 (15)	0.0178 (16)	0.0078 (15)	−0.0014 (12)	0.0033 (12)	−0.0016 (12)
C3	0.0148 (16)	0.0178 (17)	0.0074 (15)	0.0003 (13)	0.0066 (13)	−0.0011 (13)
C4	0.0149 (17)	0.0137 (16)	0.0098 (15)	−0.0011 (12)	0.0059 (13)	0.0002 (13)
C5	0.0131 (16)	0.0191 (17)	0.0054 (14)	0.0003 (13)	0.0043 (13)	0.0003 (13)
C6	0.0113 (16)	0.0179 (17)	0.0113 (15)	0.0057 (13)	0.0048 (13)	0.0021 (13)
C7	0.0144 (17)	0.0181 (17)	0.0133 (15)	−0.0016 (13)	0.0072 (14)	−0.0002 (13)
C8	0.0100 (16)	0.0173 (16)	0.0110 (16)	0.0005 (13)	0.0058 (13)	−0.0001 (13)
C9	0.0123 (17)	0.0157 (17)	0.0099 (16)	−0.0004 (12)	0.0032 (13)	0.0012 (12)
C10	0.0179 (17)	0.0166 (17)	0.0116 (15)	0.0062 (13)	0.0088 (13)	0.0015 (13)
C11	0.0176 (18)	0.0166 (16)	0.0159 (16)	−0.0035 (14)	0.0095 (14)	−0.0022 (13)
C12	0.0137 (17)	0.0227 (18)	0.0094 (16)	0.0010 (14)	0.0045 (14)	−0.0018 (13)
C13	0.0185 (18)	0.0120 (16)	0.0171 (17)	0.0014 (13)	0.0076 (14)	0.0016 (13)
C14	0.0181 (18)	0.0208 (18)	0.0161 (17)	−0.0020 (14)	0.0082 (15)	−0.0021 (14)
C15	0.0180 (18)	0.029 (2)	0.0142 (18)	0.0038 (15)	0.0078 (15)	0.0010 (15)
C16	0.0192 (19)	0.0183 (17)	0.0205 (18)	0.0005 (14)	0.0106 (16)	−0.0013 (15)
C17	0.0183 (18)	0.0180 (17)	0.0141 (17)	−0.0040 (14)	0.0089 (14)	0.0003 (14)
C18	0.0186 (19)	0.0179 (18)	0.025 (2)	0.0008 (14)	0.0128 (16)	0.0012 (15)
C19	0.028 (2)	0.0199 (19)	0.0206 (18)	−0.0080 (15)	0.0146 (17)	−0.0044 (15)
C20	0.0232 (19)	0.0206 (18)	0.0116 (16)	−0.0058 (14)	0.0070 (14)	0.0006 (14)
C21	0.0116 (17)	0.0217 (18)	0.0212 (18)	−0.0010 (13)	0.0074 (14)	−0.0010 (15)
C22	0.0175 (17)	0.0133 (16)	0.0157 (17)	−0.0064 (13)	0.0109 (14)	−0.0040 (13)
C23	0.0160 (17)	0.0167 (17)	0.0152 (17)	−0.0009 (14)	0.0067 (14)	0.0004 (14)
C24	0.0157 (17)	0.0146 (17)	0.0248 (19)	−0.0022 (14)	0.0108 (15)	0.0004 (14)

### Geometric parameters (Å, º)

**Table:**

N1—C10	1.347 (5)	C4—H4	0.9500
N1—C14	1.352 (5)	C5—C6	1.382 (5)
N1—H1A	0.8400 (12)	C5—C9	1.517 (5)
N2—C15	1.351 (5)	C6—H6	0.9500
N2—C19	1.353 (5)	C10—C11	1.363 (5)
N2—H2A	0.8400 (11)	C10—H10	0.9500
N3—C24	1.345 (5)	C11—C12	1.429 (5)
N3—C20	1.345 (5)	C11—H11	0.9500
N3—H3A	0.8400 (11)	C12—C13	1.421 (5)
O1—C7	1.325 (4)	C13—C14	1.377 (5)
O1—H1'	0.8400 (11)	C13—H13	0.9500
O2—C7	1.216 (4)	C14—H14	0.9500
O3—C8	1.311 (4)	C15—C16	1.365 (5)
O3—H3'	0.8400 (11)	C15—H15	0.9500
O4—C8	1.217 (4)	C16—C17	1.425 (5)
O5—C9	1.301 (4)	C16—H16	0.9500
O5—H5'	0.8400 (12)	C17—C18	1.429 (5)
O6—C9	1.217 (4)	C18—C19	1.369 (5)
O7—C12	1.280 (4)	C18—H18	0.9500
O8—C17	1.275 (4)	C19—H19	0.9500
O9—C22	1.288 (4)	C20—C21	1.370 (5)
C1—C2	1.394 (5)	C20—H20	0.9500
C1—C6	1.394 (5)	C21—C22	1.431 (5)
C1—C7	1.501 (5)	C21—H21	0.9500
C2—C3	1.395 (5)	C22—C23	1.421 (5)
C2—H2	0.9500	C23—C24	1.364 (5)
C3—C4	1.404 (5)	C23—H23	0.9500
C3—C8	1.492 (5)	C24—H24	0.9500
C4—C5	1.389 (5)		
			
C10—N1—C14	121.4 (3)	C10—C11—C12	119.6 (3)
C10—N1—H1A	118 (3)	C10—C11—H11	120.2
C14—N1—H1A	120 (3)	C12—C11—H11	120.2
C15—N2—C19	121.0 (3)	O7—C12—C13	121.9 (3)
C15—N2—H2A	120 (3)	O7—C12—C11	121.4 (3)
C19—N2—H2A	117 (3)	C13—C12—C11	116.7 (3)
C24—N3—C20	121.1 (3)	C14—C13—C12	120.6 (3)
C24—N3—H3A	122 (3)	C14—C13—H13	119.7
C20—N3—H3A	117 (3)	C12—C13—H13	119.7
C7—O1—H1'	110 (3)	N1—C14—C13	120.0 (3)
C8—O3—H3'	118 (3)	N1—C14—H14	120.0
C9—O5—H5'	113 (3)	C13—C14—H14	120.0
C2—C1—C6	119.8 (3)	N2—C15—C16	121.0 (3)
C2—C1—C7	121.7 (3)	N2—C15—H15	119.5
C6—C1—C7	118.4 (3)	C16—C15—H15	119.5
C1—C2—C3	119.9 (3)	C15—C16—C17	121.0 (3)
C1—C2—H2	120.0	C15—C16—H16	119.5
C3—C2—H2	120.0	C17—C16—H16	119.5
C2—C3—C4	119.9 (3)	O8—C17—C16	122.4 (3)
C2—C3—C8	119.0 (3)	O8—C17—C18	122.2 (3)
C4—C3—C8	121.0 (3)	C16—C17—C18	115.4 (3)
C5—C4—C3	119.6 (3)	C19—C18—C17	121.1 (3)
C5—C4—H4	120.2	C19—C18—H18	119.4
C3—C4—H4	120.2	C17—C18—H18	119.4
C6—C5—C4	120.5 (3)	N2—C19—C18	120.5 (3)
C6—C5—C9	120.4 (3)	N2—C19—H19	119.7
C4—C5—C9	119.1 (3)	C18—C19—H19	119.7
C5—C6—C1	120.3 (3)	N3—C20—C21	120.8 (3)
C5—C6—H6	119.8	N3—C20—H20	119.6
C1—C6—H6	119.8	C21—C20—H20	119.6
O2—C7—O1	123.8 (3)	C20—C21—C22	120.4 (3)
O2—C7—C1	122.3 (3)	C20—C21—H21	119.8
O1—C7—C1	113.9 (3)	C22—C21—H21	119.8
O4—C8—O3	124.6 (3)	O9—C22—C23	122.0 (3)
O4—C8—C3	122.8 (3)	O9—C22—C21	122.1 (3)
O3—C8—C3	112.6 (3)	C23—C22—C21	115.9 (3)
O6—C9—O5	125.1 (3)	C24—C23—C22	120.4 (3)
O6—C9—C5	122.4 (3)	C24—C23—H23	119.8
O5—C9—C5	112.5 (3)	C22—C23—H23	119.8
N1—C10—C11	121.6 (3)	N3—C24—C23	121.3 (3)
N1—C10—H10	119.2	N3—C24—H24	119.4
C11—C10—H10	119.2	C23—C24—H24	119.4
			
C6—C1—C2—C3	−0.8 (5)	C14—N1—C10—C11	−2.5 (5)
C7—C1—C2—C3	−178.1 (3)	N1—C10—C11—C12	1.4 (5)
C1—C2—C3—C4	0.3 (5)	C10—C11—C12—O7	179.9 (3)
C1—C2—C3—C8	176.4 (3)	C10—C11—C12—C13	0.3 (5)
C2—C3—C4—C5	0.2 (5)	O7—C12—C13—C14	179.5 (3)
C8—C3—C4—C5	−175.8 (3)	C11—C12—C13—C14	−1.0 (5)
C3—C4—C5—C6	−0.2 (5)	C10—N1—C14—C13	1.8 (5)
C3—C4—C5—C9	177.0 (3)	C12—C13—C14—N1	0.0 (5)
C4—C5—C6—C1	−0.2 (5)	C19—N2—C15—C16	0.5 (5)
C9—C5—C6—C1	−177.5 (3)	N2—C15—C16—C17	−1.1 (5)
C2—C1—C6—C5	0.8 (5)	C15—C16—C17—O8	−177.4 (3)
C7—C1—C6—C5	178.1 (3)	C15—C16—C17—C18	0.9 (5)
C2—C1—C7—O2	−172.4 (3)	O8—C17—C18—C19	178.1 (3)
C6—C1—C7—O2	10.2 (5)	C16—C17—C18—C19	−0.2 (5)
C2—C1—C7—O1	7.9 (5)	C15—N2—C19—C18	0.2 (6)
C6—C1—C7—O1	−169.4 (3)	C17—C18—C19—N2	−0.4 (6)
C2—C3—C8—O4	3.5 (5)	C24—N3—C20—C21	−1.5 (5)
C4—C3—C8—O4	179.5 (3)	N3—C20—C21—C22	−2.0 (6)
C2—C3—C8—O3	−176.4 (3)	C20—C21—C22—O9	−175.4 (3)
C4—C3—C8—O3	−0.4 (4)	C20—C21—C22—C23	4.8 (5)
C6—C5—C9—O6	−168.8 (3)	O9—C22—C23—C24	175.8 (3)
C4—C5—C9—O6	13.9 (5)	C21—C22—C23—C24	−4.3 (5)
C6—C5—C9—O5	11.8 (4)	C20—N3—C24—C23	2.0 (5)
C4—C5—C9—O5	−165.5 (3)	C22—C23—C24—N3	1.1 (5)

### Hydrogen-bond geometry (Å, º)

**Table:**

*D*—H···*A*	*D*—H	H···*A*	*D*···*A*	*D*—H···*A*
O1—H1′···O8^i^	0.84	1.72	2.555 (3)	173
O3—H3′···O7^ii^	0.84	1.70	2.489 (3)	156
O5—H5′···O9^iii^	0.84	1.70	2.531 (4)	170
N1—H1*A*···O7^iv^	0.84	1.91	2.712 (4)	158
N2—H2*A*···O8^v^	0.84	2.00	2.817 (4)	165
N3—H3*A*···O9^vi^	0.84	2.09	2.825 (4)	146
C14—H14···O6	0.95	2.67	3.596 (5)	166
C19—H19···O2^vii^	0.95	2.48	3.034 (5)	117
C24—H24···O4^viii^	0.95	2.45	3.067 (6)	123
C13—H13···O9^iii^	0.95	2.63	3.270 (4)	125
C10—H10···O3^ix^	0.95	2.42	3.073 (5)	126
C16—H16···O1^x^	0.95	2.68	3.307 (4)	124
C19—H19···O6^xi^	0.95	2.57	3.314 (6)	135
C20—H20···O6^xii^	0.95	2.55	3.499 (4)	176
C23—H23···O4^xiii^	0.95	2.48	3.310 (4)	147

Symmetry codes: (i) *x*−1, *y*, *z*; (ii) *x*−1, *y*, *z*−1; (iii) *x*+1/2, *y*+1/2, *z*; (iv) *x*−1/2, −*y*+3/2, *z*−1/2; (v) *x*−1/2, −*y*+1/2, *z*−1/2; (vi) *x*, *y*, *z*−1; (vii) *x*+1/2, −*y*+1/2, *z*−1/2; (viii) *x*+1/2, *y*−1/2, *z*; (ix) *x*+1/2, −*y*+3/2, *z*+1/2; (x) *x*+1, *y*, *z*; (xi) *x*+1/2, *y*−1/2, *z*−1; (xii) *x*−1/2, *y*−1/2, *z*−1; (xiii) *x*+1/2, *y*−1/2, *z*+1.
